# Supplementary material for: B-cell targeting with anti-CD38 daratumumab: implications for differentiation and memory responses
Source: Life Sci Alliance. 2023 Jul 7;6(9):e202302214. doi: 10.26508/lsa.202302214 (PMC10331639; doi:10.26508/lsa.202302214)
Supplement: Supplementary file 1 [file LSA-2023-02214_TableS1.docx]

**Table S1. List of autoimmune diseases in which daratumumab treatment has been reported to be clinically beneficial.**

| **Condition** | **References** |
| --- | --- |
| Antineutrophil cytoplasmic antibody (ANCA) -associated vasculitis | [48] |
| Anti-interferon-γ-auto-antibody mediated nontuberculous mycobacterial infection | [71] |
| Antiphospholipid syndrome | [72] |
| Auto-antibody mediated encephalitis | [73, 74] |
| Autoimmune haemolytic anaemia | [55, 75, 76] |
| Immunoglobulin light-chain amyloidosis | [77] |
| POEMS syndrome (polyradiculoneuropathy, organomegaly, endocrinopathy, monoclonal plasma cell disorder and skin changes) | [78] |
| Post-allogeneic stem cell transplantation cytopenia or red cell aplasia | [79-81] |
| Proliferative glomerulonephritis | [82] |
| Systemische lupus erythematodes (SLE) | [83] |
